# Multicomponent occupational lifestyle intervention to improve physical activity, musculoskeletal health, and work environment among Japanese teleworkers (TELEWORK study): protocol for a cluster randomized controlled trial

**DOI:** 10.1093/joccuh/uiaf014

**Published:** 2025-03-18

**Authors:** Aya Wada, Jihoon Kim, Satoru Kanamori, Takahiko Yoshimoto, Rumi Tsukinoki, Naoki Kagi, Wataru Umishio, Ryo Asaoka, Tomoko Shiomitsu, Kayo Kawamata, Natsumi Yoshioka, Kaori Yoshiba, Masahiko Gosho, Yoshio Nakata, Yuko Kai

**Affiliations:** Physical Fitness Research Institute, Meiji Yasuda Life Foundation of Health and Welfare, Tokyo 192–0001, Japan; Institute of Health and Sport Sciences, University of Tsukuba, Ibaraki 305–8574, Japan; Graduate School of Public Health, Teikyo University, Tokyo 173–8605, Japan; Department of Hygiene, Public Health and Preventive Medicine, Showa University School of Medicine, Tokyo 142–8555, Japan; Department of Public Health Nursing, Institute of Science Tokyo, Tokyo 113–8519, Japan; Department of Architecture and Building Engineering, School of Environment and Society, Institute of Science Tokyo, Tokyo 152–8552, Japan; Department of Architecture and Building Engineering, School of Environment and Society, Institute of Science Tokyo, Tokyo 152–8552, Japan; Department of Architecture and Building Engineering, School of Environment and Society, Institute of Science Tokyo, Tokyo 152–8552, Japan; Department of Public Health Nursing, Institute of Science Tokyo, Tokyo 113–8519, Japan; School of Nursing, The International University of Kagoshima, Kagoshima 891–0197, Japan; Japan Industrial Safety and Health Association, Tokyo 108–0014, Japan; Physical Fitness Research Institute, Meiji Yasuda Life Foundation of Health and Welfare, Tokyo 192–0001, Japan; Physical Fitness Research Institute, Meiji Yasuda Life Foundation of Health and Welfare, Tokyo 192–0001, Japan; Department of Biostatistics, Institute of Medicine, University of Tsukuba, Tsukuba, Ibaraki 305–8575, Japan; Institute of Health and Sport Sciences, University of Tsukuba, Ibaraki 305–8574, Japan; Physical Fitness Research Institute, Meiji Yasuda Life Foundation of Health and Welfare, Tokyo 192–0001, Japan

**Keywords:** teleworking, physical activity, musculoskeletal health, work environment, occupational health, health promotion

## Abstract

**Objectives:**

Teleworking from home was widespread during the 2019 coronavirus disease pandemic. This working practice is expected to maintain popularity among employers and employees. Compared with in-person workers, teleworkers tend to be less physically active and have more musculoskeletal pain. Interventions specific to reducing health risks among teleworkers have not been identified. This study will examine the effects of an occupational lifestyle intervention encompassing 3 components: physical activity promotion, musculoskeletal health, and work environment improvement.

**Methods:**

This cluster randomized trial will have a target sample size of 500 participants. The target population is healthy adults aged 18-64 years who telework at least once per week. Randomization will be conducted on a stratified block basis for clusters of 20 to 100 individuals within the recruited companies. The intervention period will be 12 weeks and comprise individual (online lectures, feedback, and periodic email messages), sociocultural (team building through step competition), physical (poster and tabletop pop-up), and organizational (encouraging message from an executive) strategies. The intervention group will be compared with a wait-list control group. The primary outcome will be the number of steps taken, as assessed by an accelerometer, and the secondary outcomes will be musculoskeletal pain and a telecommuting environment. The study protocol was registered with the University Hospital Medical Information Network Clinical Trials Registry (ID: UMIN000053861) (https://center6.umin.ac.jp/cgi-open-bin/ctr/ctr.cgi?function=brows&action=brows&recptno=R000061478&type=summary&language=J).

**Results:**

Study enrollment began in March 2024, and the intervention will be completed by March 2025.

**Conclusions:**

The results of this study are expected to provide helpful data for promoting healthy teleworking practices.

## Teaser

Key points

**What is already known on this topic:** The effectiveness of workplace interventions in increasing physical activity and reducing musculoskeletal pain has been established. However, no interventions specific to teleworkers that reduce their health risks have been identified.

**What this study adds:** This study proposes health risk reduction measures for teleworkers. The intervention program is based on past findings and can be implemented without difficulty among teleworkers who do not work from the office.

**How this study might affect research, practice, or policy:** If this intervention method proves to be effective, the “Guideline to Promote the Appropriate Introduction and Implementation of Telework” developed in Japan can be strengthened.

## 1. Introduction

Teleworking from home, which became widespread during the 2019 coronavirus disease pandemic, is expected to maintain popularity among employers and employees.^1^ Teleworking from home is defined as “work that takes place fully or partly within the worker’s own residence,”^2^ and has several advantages for workers, such as reduced commuting burdens and improved work-life balance.^3^ In 2019, Japan enacted a law to reform work styles and secure a diverse workforce to address a serious labor shortage.^4^ Telework is attracting attention in this context as a flexible work style. Hybrid models, such as 3 days of office work and 2 days of telework, have also been established,^5^ and the percentage of teleworking companies in Japan’s capital city of Tokyo has remained at over 40% even after the pandemic .^6^

Although teleworking from home brings about changes in workers’ daily lives, several health risks associated with these changes have been reported in recent years. The first is a decrease in physical activity (PA) due to the lack of commuting and office mobility. Teleworkers tend to be less physically active and spend more time sitting than their commuting counterparts do,^7^ a behavior that increases an individual’s risk for future lifestyle-related diseases^8^^,^^9^ and falls^10^ associated with reduced physical fitness. The second reason is the occurrence of physical symptoms associated with an inadequate work environment at home, such as eye strain and musculoskeletal pain.^11^ There is an urgent need to identify strategies to reduce these health risks and sustain teleworking with healthy lifestyles.

Interventions for these health risks can be synergistic by means of a comprehensive approach that simultaneously addresses PA, musculoskeletal health, and the work environment. Reducing sedentary behavior and increasing PA contribute to the improvement of physical symptoms such as low back pain (LBP).^12^ Considering that a poor telecommuting environment is closely associated with physical symptoms,^11^ improvement of the environment is expected to decrease physical symptoms. In participants who have physical symptoms, it is assumed that improvement in these symptoms will increase physical activity. In addition, this synergistic comprehensive approach will meet the diverse needs of workers and enhance the feasibility of interventions.

Considering that this is a workplace intervention, a multicomponent approach that addresses not only the individual level but also the environmental, organizational, and other levels is needed. Many workers are aware of the benefits of engaging in healthy behaviors, but implementation is hampered by factors such as a tightly controlled work environment and differences in perspective between employers and employees.^13^ For example, without a common understanding that staying active at work or viewing health videos is beneficial, colleagues may perceive these behaviors as counteractive to the employer’s mission. A multicomponent intervention was effective in promoting PA in the workplace based on the social-ecological model,^14^^,^^15^ a theory that indicates that human behavior is influenced not only by intraindividual characteristics but also by multilayered factors such as individuals, organizations, regions, and policies.^16^

To the best of our knowledge, there has been no approach that comprehensively addresses the health risks of teleworkers. In terms of individual approaches, previous studies that focused on PA^14^^,^^15^ considered not only the individual employee but also the office environment and the upper management of the organization. However, it is necessary to demonstrate the effectiveness of these approaches for teleworkers,^17^ who have a low frequency of office attendance and face-to-face communication with colleagues and supervisors. Previous studies assessing improvements to the physical environment have reported the effect on musculoskeletal health.^18^ This approach was in the office environment, whereas no studies have examined the approach in the telework environment. The only study that has conducted ergonomics training for teleworkers^19^ had a limited number of participants and study period. Therefore, a high-quality study focused on improving telecommuting environments is required.

### 1.1. Objectives

This study aims to examine the effects of an occupational lifestyle intervention encompassing 3 components—PA promotion, musculoskeletal health, and work environment improvement—among Japanese teleworkers. The 12-week intervention program comprises individual (online lectures, feedback, and periodic email messages), sociocultural (team building through step competition), physical (poster and tabletop pop-up), and organizational (encouraging message from an executive) strategies. The primary hypothesis of the study is that the intervention group will have a significantly greater change in step count over 12 weeks than the control group. The secondary outcomes will be changes in other PA parameters, physical symptoms such as LBP, and the telecommuting environment.

### 1.2. Trial design

The proposed study is a 2-arm cluster-randomized controlled trial (cRCT) with a wait-list control group. After enrollment, each cluster (workplace) will be assigned to the intervention or control group in a 1:1 ratio using stratified block randomization. The effectiveness of the intervention program will be assessed by evaluating data from the intervention and control groups for the first 12 weeks at the employee level. The control group will not be provided with any program elements during this period. However, they will be provided with the program after the waiting period, and data from this program period will be used, along with data from the intervention group, for explanatory analyses.

## 2. Methods

### 2.1. Participants

This study began in March 2024 in the Japanese workplace and will continue until March 2025. The eligibility criteria for employees are: (1) being aged ≥18 years and <65 years; and (2) being home-based teleworkers for at least 1 d/wk. The exclusion criteria for employees are: (1) being prohibited from exercising by their doctors; (2) planning long-term business trips or retirement during the study; (3) being pregnant or possibly pregnant; and (4) being judged by the research director to be inappropriate for inclusion, for example, not agreeing to return the measuring instruments. Recruitment will be conducted through the research team’s network of companies that employ teleworkers. The employee coordinator of the company that agrees to participate will send invitations to employees who meet the eligibility criteria. Based on the employees who agree to participate at this stage, clusters of 20 to 100 participants will be selected and allocated to units, such as departments and offices, where contamination does not occur. Subsequently, participants will receive baseline survey instructions and instruments.

### 2.2. Interventions

**Figure 1:**
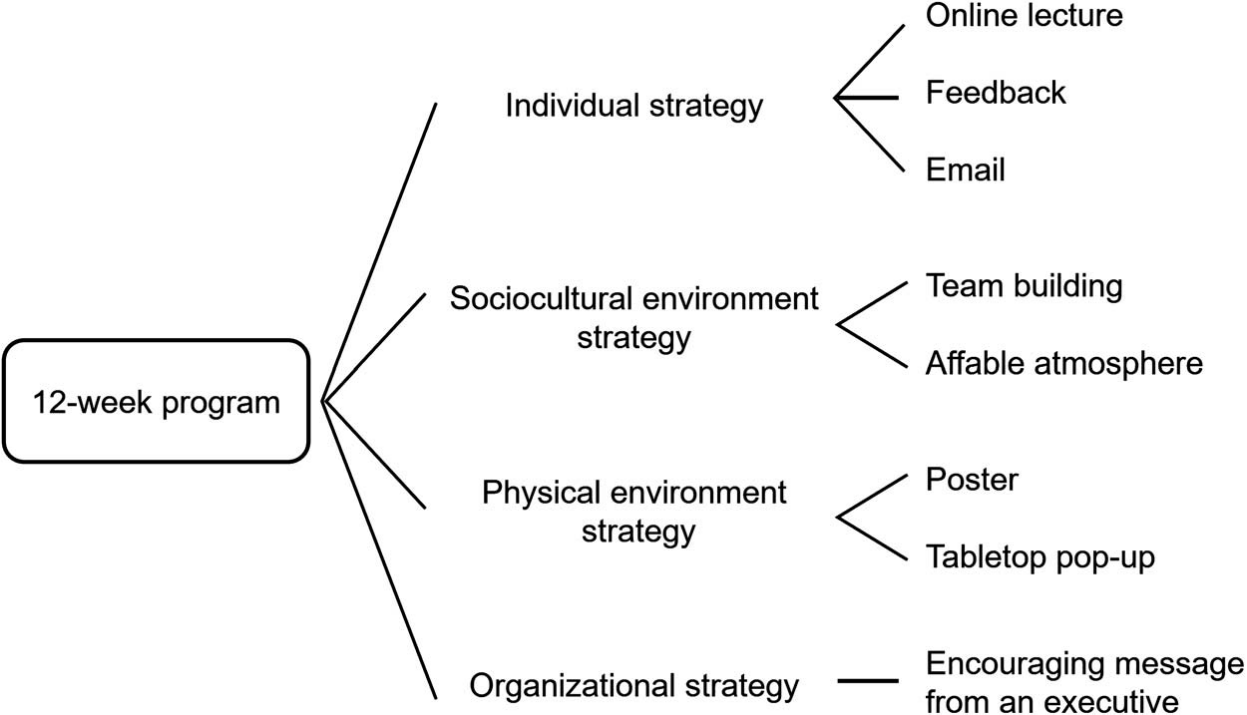
Multicomponent occupational lifestyle intervention.

The 12-week multicomponent occupational lifestyle intervention will be provided using a remote system (Figure 1). The program focuses on 3 topics: improving PA, musculoskeletal health, and the work environment. The intervention program comprises individual, sociocultural environment, physical environment, and organizational strategies based on the social-ecological model.^14^^-^^16^ We plan to collaborate with an employee coordinator at each site to ensure the successful delivery and implementation of the intervention. Additionally, the intervention can be tailored to each workplace’s specific circumstances (eg, in cases where some programs cannot be introduced). The implementation of other health promotion programs by each company during the study period will be generally permitted.

#### 2.2.1. Individual strategy

Individual strategies include online lectures, feedback, and email messages. First, we established a website where participants can access online lectures about improving PA, musculoskeletal health, and their work environment at their convenience from any location.

The PA section consists of 3 theoretical lectures (4-5 minutes each) and specific exercise movies (2-5.5 minutes each). The researcher (Y.N.) will deliver theoretical lectures on PA promotion. The content will include definitions of PA and sedentary behavior, the impact of physical inactivity and sedentary behavior on health, and evidence of “+10 minutes of PA per day.”^20^ The lectures on specific exercise methods contain five 3-minute stretching exercises (produced by Science Tokyo and Nomura Real Estate Group) focused on locomotion training for workers.^21^ In the musculoskeletal health section, we will provide 7 short videos of exercises (1.5-4 minutes each) and 5 videos of evidence-based information on LBP (2-4 minutes each). Exercise programs consist of muscle stretching and strengthening to improve postural alignment and spinal stability. The exercise menu comprises 7 therapeutic self-exercises, including standing back extension “One stretch,”^22^ trunk lateral flexion, sitting trunk extension, seated hamstring stretch, lying trunk twisting, lunge exercise, and arm-leg raising. Educational videos comprise 5 tips with information on LBP management (staying active, importance of self-management, fear-avoidance belief, psychological stress, and no need to be concerned about spine imaging findings). Matsudaira’s research team created these educational videos with reference to several clinical guidelines and media campaigns for LBP.^23^ We will also provide pamphlets on LBP management, which include biomechanical (eg, posture, exercise) and psychosocial aspects (eg, psychological stress at the workplace and fear-avoidance beliefs).

In the work environment section, 5 videos were created by one researcher (S.K.) on improving the telework environment at home (2-4 minutes each). The content of each video covers 9 themes and 12 environmental improvement actions, referring to previous studies,^11^ a technical brief,^24^ a checklist,^25^ and others. The content covers topics such as illumination and displays, keyboards and mice, feet and chairs, temperature, humidity, air quality, and noise. These videos include several behavioral science findings to inspire action. For example, quizzes will be administered. Information-gap theory, which states that people are intrigued by what they do not understand, has been referenced.^26^

As feedback, participants in the intervention group will receive an occupational health report about 3 topics. The PA report will include daily step counts; light, moderate, vigorous, and moderate-to-vigorous PA that has been undertaken; sedentary time; prolonged sedentary time; and recommended standards. All of these figures will be provided separately for teleworking days and office days. The musculoskeletal health report will include LBP pain intensity (based on a numerical rating scale, 0-10). The work environment report will include environmental sensor measurements (temperature, relative humidity, illuminance, and noise level) and their recommended ranges. Additionally, an evaluation of telework environment improvement actions will be presented. This evaluation will be based on telework environment improvement behavior in the outcomes of this study.^11^^,^^24^^,^^25^

The intervention group will be provided with email messages twice a week—on Tuesdays and Fridays—for 12 weeks. The content of the messages will include requests for active participation, recommendations based on baseline surveys, PA promotion, LBP prevention tips, and work environment improvement methods. The research team will send the messages to the intervention group’s coordinator, who will then email them to the participants. To improve adherence in companies with joint meetings, researchers will ask their supervisors to conduct the exercises that were most recently distributed via email in a group setting. The frequency of implementation will be measured in a post-survey. Employees who will not participate in the study may not be prohibited from receiving the intervention program in part.

#### 2.2.2. Sociocultural environment strategy

The intervention group will undertake a step competition to create an atmosphere that encourages participants to actively engage in the program. This competition will include individual and team components, with the results announced through weekly messages.

#### 2.2.3. Physical environment strategy

The intervention group will receive posters aimed at promoting PA. These posters will be accessible on a notice board in the office or on a website. The topics will include the health risks associated with prolonged sitting, health benefits of replacing sitting with standing, health benefits of walking, and importance of goal-setting for improving PA. Posters will be replaced with new posters every 3 weeks.

The pamphlet on LBP management mentioned above will include a tabletop pop-up that individuals can easily create. The tabletop pop-up will show how to do “One stretch,” with illustrated instructions. We will encourage the intervention group to create the tabletop pop-up to place on their work desk when we deliver the pamphlet.

#### 2.2.4. Organizational strategy

As an organizational strategy, encouraging messages from upper management will be provided to the participants in the intervention group. The content of the mailings will be determined by each upper management, but will include the importance of participation and specific encouragement based on baseline survey results.

### 2.3. Outcomes

#### 2.3.1. Physical activity

The primary outcome is the change in daily step count between baseline and the end of the intervention. The secondary outcomes include changes in light, moderate, vigorous, and moderate-to-vigorous PA; sedentary time; and prolonged sedentary time. Participants will be asked to wear a triaxial accelerometer (Active-style Pro HJA-350IT; Omron Healthcare, Kyoto, Japan) on their waist for 14 days.^27^^,^^28^ For safety reasons, participants will be requested to remove the device when sleeping, bathing, swimming, or playing contact sports. Additionally, participants will maintain a daily diary of whether they were working, their working time, and the type of work they were doing (remote or commuting). Finally, we will use valid accelerometer data (at least 10 h/d^29^ and a minimum of 3 valid days) in the analysis.

#### 2.3.2. Musculoskeletal health

The outcomes of the musculoskeletal health section will include the intensity of LBP (numerical rating scale, 0-10), degree of disability due to LBP, attitude/belief about LBP, work productivity, subjective improvement in LBP, and frequency of exercise performed (individual/group). Disability due to LBP will be indicated according to 4 grades: grade 0 (no LBP) to grade 3 (LBP interfering with work and leading to sick leave), with a modification of Von Korff’s grading method.^30^ Attitude/belief about LBP will be assessed using the 5 statements about fear-avoidance beliefs and passive coping strategies on a 4-point Likert scale (1 = strongly disagree, to 4 = strongly agree). Work productivity will be assessed using the presenteeism item in the LBP version of Work Productivity and Activity Impairment (WPAI),^31^ and subjective improvement in LBP will be assessed using the Patient’s Global Impression of Change (PGIC).^32^ Participants will be asked, “Since the start of the study, how has your LBP changed?” The options will be rated on a 7-point Likert scale (1 = very much improved, to 7 = very much worse).

#### 2.3.3. Work environment

The telecommuting environment will be assessed objectively and subjectively. The objective telework environment will be measured on the worker’s desk at 10-minute intervals using an environmental sensor (2JCIE-BL01; Omron, Kyoto, Japan). The measurement parameters will include temperature, relative humidity, illuminance, and noise level. For comparison purposes, measurements will be conducted at representative points in the office in the same manner. For the analysis, teleworking environment data during the working hours of each worker will be extracted based on a diary survey. Office environment data will be extracted from 9:00 am to 5:00 pm (excluding midday to 1:00 pm) on weekdays, considering regular work time.

Subjective telework and office environments will be assessed by workers using the Subjective Assessment of workplace Productivity (SAP) questionnaire.^33^ The workers’ satisfaction with the telework and office environments (including lighting, thermal, air, sound, spatial, and IT environment) will be assessed on a 5-point Likert scale (1 = dissatisfied, to 5 = satisfied). Additionally, whether the respondent has taken action to improve the telecommuting environment will also be evaluated. As there is no existing scale for this assessment item, a 12-behavior questionnaire was created based on previous research,^11^ a technical brief,^24^ a checklist,^25^ and others. Each item will be rated on a 5-point Likert scale (1 = not applicable, to 5 = applicable).

Improvements in these teleworking-environment behaviors are expected to improve somatic symptoms.^11^ Therefore, the Somatic Symptom Scale-8 (SSS-8)^34^ will be used to evaluate somatic symptoms for the secondary outcomes.

#### 2.3.4. Work-related and psychological outcomes

Work productivity and psychological status, estimated to be affected by improvements in PA and physical symptoms, will be assessed. Participants’ presenteeism will be assessed using the Japanese version of the World Health Organization Health and Work Performance Questionnaire (WHO-HPQ).^35^ Work engagement will be measured using the Japanese version of the Utrecht Work Engagement Scale (UWES-9), which evaluates 3 dimensions: vigor, dedication, and absorption.^36^ For psychological outcomes, mood and anxiety disorders will be measured using selected questions from the Japanese version of the Kessler Psychological Distress Scale (K6).^37^

#### 2.3.5. Process evaluation after the intervention

The degree of participation and satisfaction (eg, whether the participants consumed each piece of content, whether they took informed action, their impressions of the frequency of delivery and content, and whether there was an atmosphere of workplace-wide commitment and satisfaction) will be surveyed after the intervention. An interview will also be conducted with some of the study participants after the intervention to assess the feasibility and effectiveness of, and barriers to, the program. The interviews will be conducted using a qualitative research design with focus groups.

### 2.4. Participant timeline

Table 1 presents the schedule of enrollment, interventions, and assessments for this cRCT. Enrollment is scheduled between March and July 2024. Outcomes will be measured during the 2 weeks before and after the intervention.

**Table 1:** Schedule of enrollment, interventions, and assessments.

	**STUDY PERIOD**						
	**Enrollment**	**Allocation**	**Post-allocation**				
**TIMEPOINT**	**March-July 2024**	**Immediately after enrollment**	**Baseline**		**3-month follow-up**		**6-month follow-up (only CG)**
**ENROLLMENT:**							
**Eligibility screen**	X						
**Informed consent**	X						
**Allocation**		X					
**INTERVENTIONS:**							
**Intervention group (IG)**				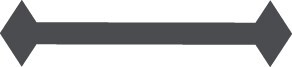			
**Control group (CG)**						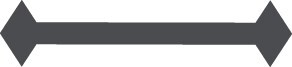	
**ASSESSMENTS:**							
**PA (step count; light, moderate, vigorous, and moderate-to-vigorous PA; sedentary time; and prolonged sedentary time)**			X		X		X
**Intensity of LBP**			X		X		X
**Disability due to LBP**			X		X		X
**Attitude/belief about LBP**			X		X		X
**Work productivity and activity impairment**			X		X		X
**Subjective improvement of LBP (PGIC)**					X		X
**Frequency of exercise**			X		X		X
**Work environment (temperature, relative humidity, lighting, and noise level)**			X		X		X
**Satisfaction with work environments (SAP)**			X		X		X
**Action to improve the telecommuting environment**			X		X		X
**Somatic symptoms (SSS-8)**			X		X		X
**Presenteeism (WHO-HPQ)**			X		X		X
**Work engagement (UWES-9)**			X		X		X
**Psychological outcomes (K6)**			X		X		X
**Participation and satisfaction**					X (only IG)		X
**Feasibility of and barriers to the program (interview)**					X (only IG)		

Abbreviations: CG, control group; IG, intervention group; K6, Kessler Psychological Distress Scale; LBP, low back pain; PA, physical activity; PGIC, Patient’s Global Impression of Change; SAP, Subjective Assessment of workplace Productivity; SSS-8, Somatic Symptom Scale-8; UWES-9, Utrecht Work Engagement Scale; WHO-HPQ, World Health Organization Health and Work Performance Questionnaire.

### 2.5. Sample size

The effect size of the intervention program regarding the primary outcome, a change in step count over 12 weeks, was estimated with Cohen *d* = 0.33. This is based on a previous study,^14^ with an SD of 2400 and an estimated difference between groups of 800 in the expected increase in step count. An increase of 800 steps is also a clinically meaningful difference.^38^ The calculation was performed with a type 1 error rate of 0.05 and a statistical power of 0.80, and the required sample size was estimated at 146 in each group for a total of 292 participants. To consider the design effect as a cRCT, the average number of cluster sizes was assumed to be 20, the intraclass correlation coefficient was 0.02, and the number of participants was calculated to be 403. Additionally, we estimated that 80% of the participants would finish the intervention; therefore, a total of 500 participants would be recruited. To improve the participation rate, some of the workplace-based results will be fed back to the employee coordinator in charge of each cluster for their health promotion strategies after the study is completed.

### 2.6. Assignment of interventions

Stratified block randomization will be employed, and stratification will be based on the number of participants (<20 vs ≥20). Each cluster will be assigned to the intervention or control group based on a computer-generated random number table, which will be created by a biostatistician at the University of Tsukuba (M.G.). It will be maintained by a researcher not associated with the research team at the Physical Fitness Research Institute, Meiji Yasuda Life Foundation of Health and Welfare. The allocation will be performed through central registration and disclosed to the participants and researchers via email. Due to the nature of the intervention, blinding was considered difficult to implement.

### 2.7. Data collection and management

Self-reported data will be obtained using a web-based questionnaire, whereas objective data will be obtained using measuring instruments that will be sent to each participant. The researchers will ensure that the web-based questionnaire can be accessed from the participant’s personal computer, and easy-to-follow instructions will be attached to the measurement device. The survey period will be 2 weeks, and a reminder email will be sent to participants who do not respond. To protect the privacy of personal information, the researchers will outsource email distribution and instrumentation to an investigation agency that has signed a confidential disclosure agreement. The research team will not obtain the participants’ personal information. The investigation agency will maintain a strict list of the participants’ personal information and research IDs. The investigation agency (identified data) and the research team (de-identified data) will password-protect the data and store it on a secured computer. The final trial dataset will be accessible to the researchers on the study team. However, access will be strictly managed until the trial is completed.

### 2.8. Statistical methods

Efficacy will be analyzed using an intent-to-treat approach, regardless of adherence. The characteristics of the clusters and participants will be compared by group allocation using means (SDs) or medians (interquartile ranges) for continuous variables, and counts and percentages for nominal variables. The primary outcome, which is the change in daily step count between baseline and the end of the intervention, will be analyzed using generalized estimating equations (GEEs) to adjust for clustering. The GEE method will employ an exchangeable correlation structure and an identity link with a normal distribution. The mean model will include the allocated group as a factor and baseline step count as a covariate. We will analyze secondary outcomes (PA measures, LBP scale, and work environment scale) using the same method. A sensitivity analysis will be conducted for the primary outcome based on the per protocol set. All statistical tests will be 2-sided with a 5% significance level.

### 2.9. Ethics and dissemination

The study protocol was approved by the Ethical Committee of the Meiji Yasuda Life Foundation of Health and Welfare (approval no. 2023–0002) on February 6, 2024. The study will be conducted in accordance with the ethical standards of the World Medical Association’s Helsinki Declaration (1964, amended most recently in 2013), and informed consent will be obtained from all the participants according to the following procedure. If the company agrees to participate in the study, the company leader will invite employees who meet the eligibility criteria to participate. The investigation agency will mail a consent form to each employee, who will informally consent at the time of the baseline survey, and the participant will sign and return the form officially. A privacy concern specific to teleworkers is that home addresses are obtained to post instruments to those who do not come to the office. This point will be adequately explained to the participants, and their personal information will be strictly managed. The study results will be submitted and published in a peer-reviewed scientific journal. The research team plans to publish these results as a health management guide for teleworkers.

## 3. Discussion

To the best of our knowledge, this intervention study is the first large cRCT to comprehensively target various health risks among teleworkers. The study’s rigorous cRCT design, grounding in previous research, combining subjective and objective assessments, and dissemination potential are its strengths. Contamination is difficult to avoid in workplace interventions.^39^ The cRCT design minimizes this risk. In addition, various approaches to the intervention build on previous findings. In a previous multicomponent PA promotion intervention for office workers, the participants’ (*n* = 50) PA levels were significantly improved.^14^ The encouraging messages from supervisors used in this intervention and effective in promoting health behaviors^40^ will be employed in this study. The outcome evaluation will include both objective and subjective assessments. In our study, accelerometer-obtained measurements will be used to accurately record details of daily PA patterns. The telecommuting environment is measured using environmental sensors, and little effort has been made to assess physical environments rather than IT environments. Our study is expected to capture work environments that are difficult for workers to recognize accurately. Finally, we designed a program that can be implemented by companies introducing teleworking without commuting, focusing on online lectures and email deliveries. Additionally, given the comprehensive approach, the content can easily meet the needs of many companies.

However, the generalizability of this study needs to be carefully considered because teleworkers come from diverse backgrounds, such as industry and employee size. The intervention is a program based on online lectures and emails that focuses on decreasing physical inactivity due to a lack of commuting and on reducing the health risks associated with performing visual display terminal (VDT) work at home. Because of these facts, we assume that the results of this study can be generalized to workers who have a workday without commuting and who perform VDT work at home. However, recruitment for cRCTs is difficult to fully randomize, and the limitations of the generalizability of the final results should be recognized.

The study has other limitations. The first is the possibility that nonblinding may affect the results, and the second is that long-term effects cannot be assessed. These are issues to be explored in the future. However, considering that effective measures to reduce the health risks to teleworkers are unclear, we are confident that the results of this study will provide important insights for improving occupational health.

## Supplementary Material

Web_Material_uiaf014
